# Supplementary material for: Complexome profiling of the Chlamydomonas psb28 mutant reveals TEF5 as an early PSII assembly factor
Source: Plant Cell. 2025 Mar 17;37(6):koaf055. doi: 10.1093/plcell/koaf055 (PMC12164590; doi:10.1093/plcell/koaf055)
Supplement: koaf055_Supplementary_Data [file koaf055_supplementary_data.zip › psb28_interactive.zip!/plots/intensity/qualityPlots/peptides/CPLD44_Cre17.g721700.t1.2.html]

### Cre17.g721700.t1.2

Trivial name: CPLD44  
SeqCoverage [median]: 22.60  
Mass: 25.68 kDa  
  
 

| Sequence | MissedCleavage | Mass [Da] | wtIntensity | muIntensity | Score |
| --- | --- | --- | --- | --- | --- |
| ATMVEANLK | 0 | 975.51 | 918.39 | 4607.17 | 151.50 |
| DFSNQDLR | 0 | 993.45 | 286.35 | 0.00 | 68.92 |
| DFSNQDLRR | 1 | 1149.55 | 342.15 | 2168.76 | 39.02 |
| TLPVYRN | 1 | 861.47 | 1176.91 | 3043.16 | 80.38 |
| TNFEDANLSDVLMDR | 0 | 1738.78 | 583.72 | 4098.05 | 150.21 |
| YASGTNPVTGADTR | 0 | 1408.66 | 0.00 | 1997.77 | 138.94 |
|  |  |  | 3307.5 | 15914.9 |  |
